# Accuracy of ICD-10 coding for identifying immunoglobulin A nephropathy (IgAN) prior to 2023

**DOI:** 10.1093/ckj/sfaf327

**Published:** 2025-10-23

**Authors:** Alesya P Sumantri, Carolyn K Kim, Qiaoling Chen, Nancy T Cannizzaro, John J Sim

**Affiliations:** Department of Research and Evaluation, Kaiser Permanente Southern California, Pasadena, CA, USA; Division of Nephrology and Hypertension, Kaiser Permanente Los Angeles Medical Center, Los Angeles, CA, USA; Department of Research and Evaluation, Kaiser Permanente Southern California, Pasadena, CA, USA; Department of Research and Evaluation, Kaiser Permanente Southern California, Pasadena, CA, USA; Department of Research and Evaluation, Kaiser Permanente Southern California, Pasadena, CA, USA; Division of Nephrology and Hypertension, Kaiser Permanente Los Angeles Medical Center, Los Angeles, CA, USA; Department of Clinical Science, Kaiser Permanente Bernard J. Tyson School of Medicine, Pasadena, CA, USA

To the Editor,

Immunoglobulin A nephropathy (IgAN) is one of the most common glomerular diseases worldwide and a leading cause of end-stage kidney disease (ESKD) [1]. Recent evidence has challenged the long-held perception that IgAN is a benign condition. Long-term cohort studies have demonstrated that a substantial proportion of patients with IgAN progress to kidney failure or death [2, 3]. Currently there are no validated diagnostic or prognostic markers, and diagnosis is by kidney biopsy. Accurate identification of IgAN is critical for epidemiologic research and assessing the impact of emerging therapies. Until 1 October 2023, the International Classification of Diseases, Tenth Revision (ICD-10) lacked a specific code for IgAN, forcing clinicians to rely on non-specific codes such as N02.8 and N02.9, defined as ‘recurrent and persistent haematuria with (N02.8) vs without (N0.2.9) other morphologic changes’. This limitation has hindered the use of claims data for identifying confirmed IgAN cases. ICD coding offers a powerful opportunity to identify and track rare diseases and conditions across large health systems and administrative databases, enabling more accurate epidemiologic research and targeted population health strategies [4, 5].

We conducted a cross-sectional study within Kaiser Permanente Southern California (KPSC) between 1 January 2019 and 31 December 2021. Adult members (≥18 years) with two or more separate clinical encounters coded with ICD-10 code N02.8 were identified. From this cohort, 300 patient charts (100 per calendar year) were randomly selected for manual chart review to assess for documented evidence of kidney biopsy confirming IgAN and/or clinical documentation of an IgAN diagnosis. Similarly, adult members with two or more separate encounters coded with ICD-10 code N02.9 were identified and 120 charts were randomly selected for review using the same criteria (Supplemental Figure 1). Positive predictive values (PPVs) were calculated for each code based on biopsy-confirmed or clinically documented IgAN.

Among patients coded with N02.8, 87% had biopsy-confirmed IgAN and 99% had either a biopsy or clinical documentation (PPV 0.99; Table 1). Thus, clinically diagnosed IgAN without reference to a biopsy was present in 12% of our sample. A total of 4 (1%) patients had no clinical documentation for IgAN. These reasons included a documented ‘history of IgAN’ without a nephrology visit/referral, IgAN in differentials for proteinuria/haematuria without a biopsy, presumed history of IgAN during youth with no nephrology visit/referral or kidney biopsy, and renal biopsy demonstrating acute tubular necrosis and acute interstitial nephritis without evidence of IgAN. In contrast, only 18 (15%) patients who were coded N02.9 had clinical suspicion for IgAN (PPV 0.15) and none had biopsy confirmation. These findings support using N02.8 as a valid proxy for identifying IgAN in retrospective electronic health record (EHR) studies, while N02.9 lacks specificity.

**Table 1:** Chart review of patients who had two separate encounters coded N02.8 (*n* = 300).

Chart review findings	Yes, *n*	No, *n*	Total, *N*	PPV, %
Kidney biopsy demonstrating IgAN	260	40	300	87
Clinical documentation of IgAN	296	4	300	99

IgAN has a highly heterogeneous presentation and clinical course, with broad clinicopathologic overlap within the diagnoses of primary and secondary glomerulonephritis, systemic IgA vasculitis and atypical forms. Thus accurate diagnosis and documentation are crucial for medical management given emerging therapies aimed at pathogenic antibody reduction and complement inhibition. This validation of ICD-10 code N02.8 has important implications for health systems aiming to track disease burden and evaluate treatment outcomes. Accurate coding enables better population health management and supports research for therapeutic strategies for IgAN. It is noted that ICD-10 code N02.B for ‘recurrent and persistent immunoglobulin A nephropathy’ became effective as of 1 October 2023.

Limitations include the single health system setting and reliance on retrospective documentation. Nonetheless, our findings suggest that ICD-10 code N02.8 can be reliably used to identify IgAN in EHR-based research.

## Supplementary Material

sfaf327_Supplemental_File
