# Supplementary figures and images for: Accuracy of ICD-10 coding for identifying immunoglobulin A nephropathy (IgAN) prior to 2023
Source: Clin Kidney J. 2025 Oct 23;18(11):sfaf327. doi: 10.1093/ckj/sfaf327 (PMC12605792; doi:10.1093/ckj/sfaf327)

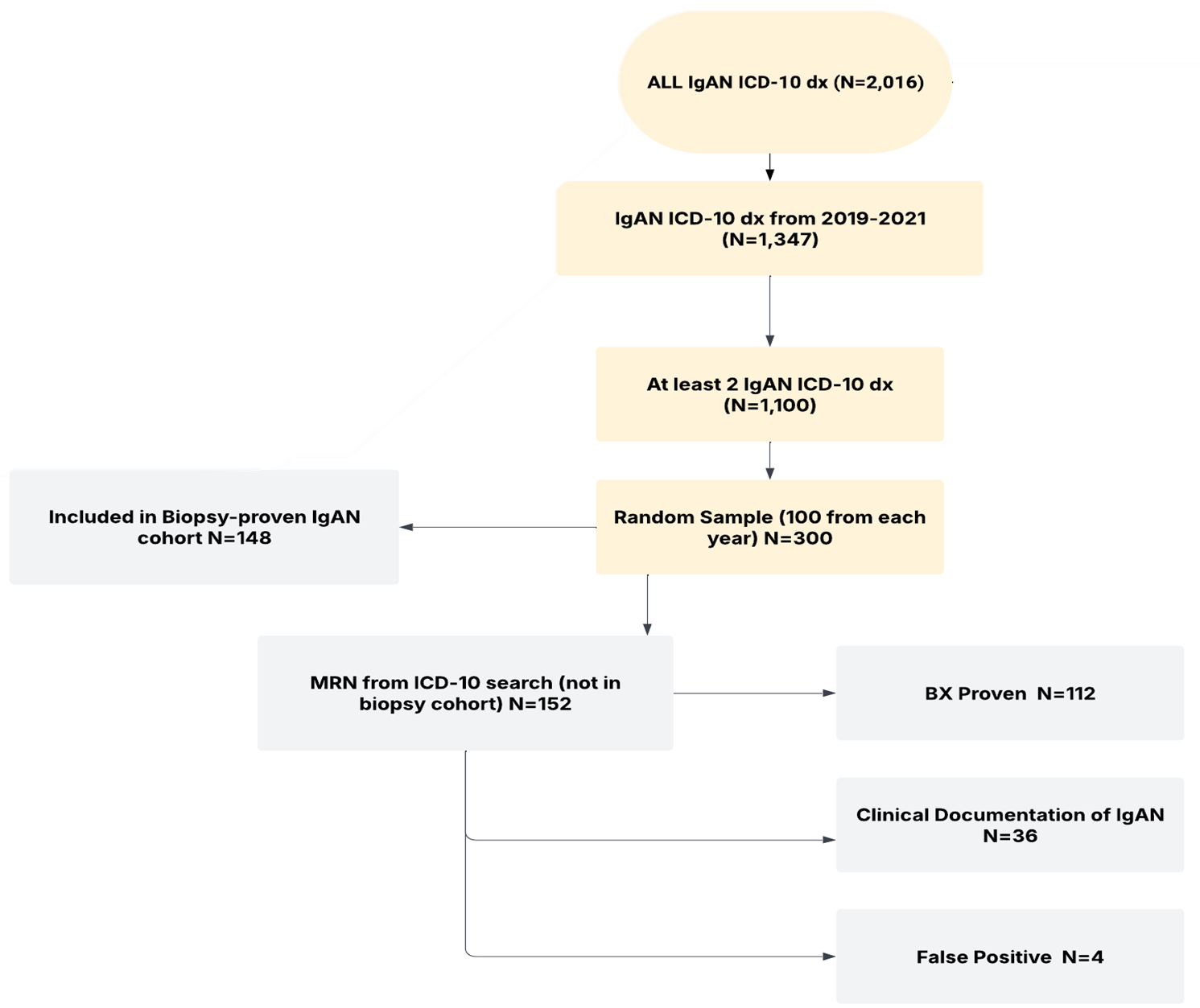
Supplemental Figure 1

Supplement: sfaf327_Supplemental_File [file sfaf327_supplemental_file.docx]
